# Light-Triggered Cargo Loading and Division of DNA-Containing Giant Unilamellar Lipid Vesicles

**DOI:** 10.1021/acs.nanolett.1c00822

**Published:** 2021-07-12

**Authors:** Yannik Dreher, Kevin Jahnke, Martin Schröter, Kerstin Göpfrich

**Affiliations:** † Max Planck Institute for Medical Research, Biophysical Engineering Group, Jahnstraße 29, 69120 Heidelberg, Germany; ‡ Department of Physics and Astronomy, Heidelberg University, 69120 Heidelberg, Germany; § Max Planck Institute for Medical Research, Department of Cellular Biophysics, Jahnstraße 29, 69120 Heidelberg, Germany

**Keywords:** Giant unilamellar lipid vesicle (GUV), synthetic cell division, lipid peroxidation, cargo loading, Chlorin e6 (Ce6)

## Abstract

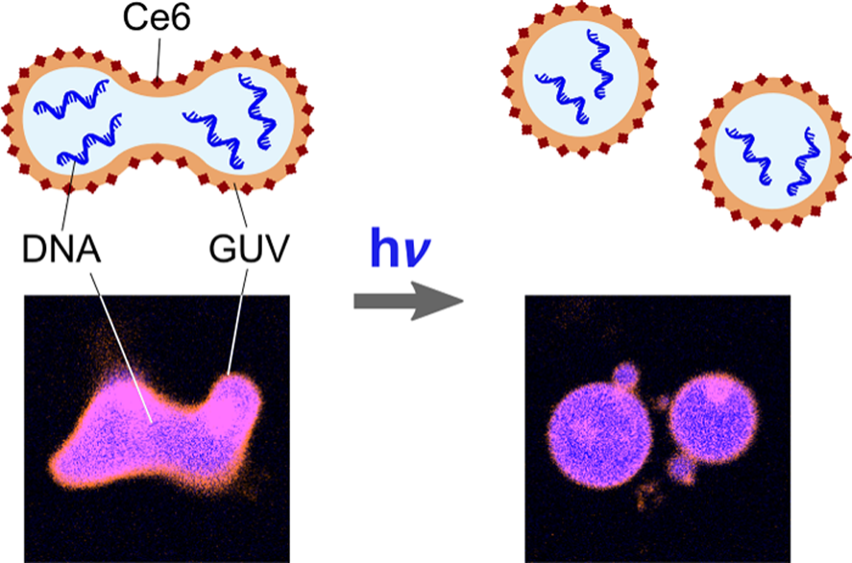

A minimal synthetic cell should contain a substrate for information storage and have the capability to divide. Notable efforts were made to assemble functional synthetic cells from the bottom up, however often lacking the capability to reproduce. Here, we develop a mechanism to fully control reversible cargo loading and division of DNA-containing giant unilamellar vesicles (GUVs) with light. We make use of the photosensitizer Chlorin e6 (Ce6) which self-assembles into lipid bilayers and leads to local lipid peroxidation upon illumination. On the time scale of minutes, illumination induces the formation of transient pores, which we exploit for cargo encapsulation or controlled release. In combination with osmosis, complete division of two daughter GUVs can be triggered within seconds of illumination due to a spontaneous curvature increase. We ultimately demonstrate the division of a selected DNA-containing GUV with full spatiotemporal control—proving the relevance of the division mechanism for bottom-up synthetic biology.

The field of bottom-up synthetic biology pursues the exciting challenge to create cell-like compartments that exhibit features and functions of living cells.^1−4^ The capability to produce offspring and to divide into two or more compartments is of major interest since it provides the basis toward a self-sustained cellular system which can ultimately evolve. Lipid membrane vesicles, in particular giant unilamellar vesicles (GUVs), have been a popular choice for synthetic cellular compartments as they mimic the cell membrane and can be engineered in diverse ways.^5−9^ The incorporation of a minimal set of proteins of the division machinery of cells into GUVs is insightful but it has not yet achieved synthetic cell division.^10−13^ One of the main challenges for GUV division is to overcome the energy barrier for the neck fission of the daughter compartments. Here, biophysical approaches have proven to provide a shortcut toward synthetic cell division. It has been known for decades that GUV morphologies depend on the surface-to-volume ratio, which can be controlled by osmosis.^14^ While osmosis can lead to deformed GUVs and the formation of buds,^15^ only a few cases of division including full neck fission have been reported so far. One approach uses the line tension at the domain boundary of phase-separated GUVs, which proved to be sufficient to overcome the energy barrier for fission and thus to achieve division at a precisely predictable osmolarity ratio.^16^ Alternatively, fission without phase separation was demonstrated relying on an increase of the spontaneous curvature for multilamellar vesicles^17−19^ and even for GUVs.^20^ Furthermore, division was achieved mechanically using a microfluidic splitting device.^21^

Nevertheless, a division mechanism which combines several of the following features would be highly desirable for bottom-up synthetic biology, but remains hitherto unachieved: 1) GUV division should be compatible with standard lipid mixtures; 2) Division should be stimuli-responsive; 3) Ideally, the division mechanism should be compatible with a loading step, such that the components required for a semiautonomous synthetic cell-cycle can be supplied via a feeding bath prior to division; and 4) On the route toward synthetic cells, it would be important to combine division with information propagation. The latter entails the division of DNA-containing GUVs, since previous accounts of division deal with empty GUVs^16,20^ or multilamellar vesicles.^19^

Here, we demonstrate the light-triggered division of DNA-containing GUVs with full spatiotemporal control. Our approach is based on osmotic deflation in combination with an increase of spontaneous curvature due to asymmetric lipid peroxidation induced by the membrane-active photosensitizer Chlorin e6 (Ce6).

The division of GUVs requires a sufficiently high surface-to-volume ratio combined with a mechanism to overcome the energy barrier for fission. Based on these requirements, we develop a division mechanism as illustrated in Figure 1A which combines several of the desirable features for synthetic biology. As a first step, the GUVs are osmotically deflated to provide sufficient excess membrane area for division into two or more compartments. In the second step, the energy barrier for fission has to be overcome. Here, we rely on an increase in spontaneous curvature. In order to gain control over the spontaneous curvature increase, we repurpose the photosensitizer Ce6. Ce6 is a low-cost compound which can be isolated from *Chlorella ellipsoidea* algae as a renewable source in large quantities. In mouse and rat models, Ce6 has been shown to lead to apoptosis of cancer cells when injected into the tumor tissue with subsequent laser illumination.^22,23^ This makes Ce6 a promising tool for photodynamic cancer therapy. When added to cells, Ce6 incorporates into the outer leaflet of cell membrane and its pharmacological mode of action relies on photoinduced oxidative stress.^24^ Ce6 illumination leads to peroxidation of unsaturated lipids in the cell membrane, which induces lipid desorption and thus pore formation on an illumination time-scale of minutes.^25^ The process that we exploit for GUV division, however, happens on a time scale of seconds: Reactive oxygen species (ROS) are generated in close proximity to the lipid tails as illustrated in Figure 1B.^26^ The ROS, in turn, induce the formation of a hydrophilic hydroperoxy-group adjacent to the double bond^27^ in the outer bilayer leaflet within seconds. At this point, the peroxidized lipids lead to an asymmetric area increase and thus an increase in the spontaneous curvature of the GUV membrane. Thus, we expect that addition of Ce6 to deflated GUVs and subsequent illumination increases the spontaneous curvature, which helps to overcome the energy barrier for neck fission. It should thus be possible to achieve complete division of GUVs into two daughter compartments. The use of light to induce the division process provides the unique advantage to select single vesicles from a bulk solution and to monitor the complete division process while the surrounding GUVs remain unaffected.

**Figure 1:**
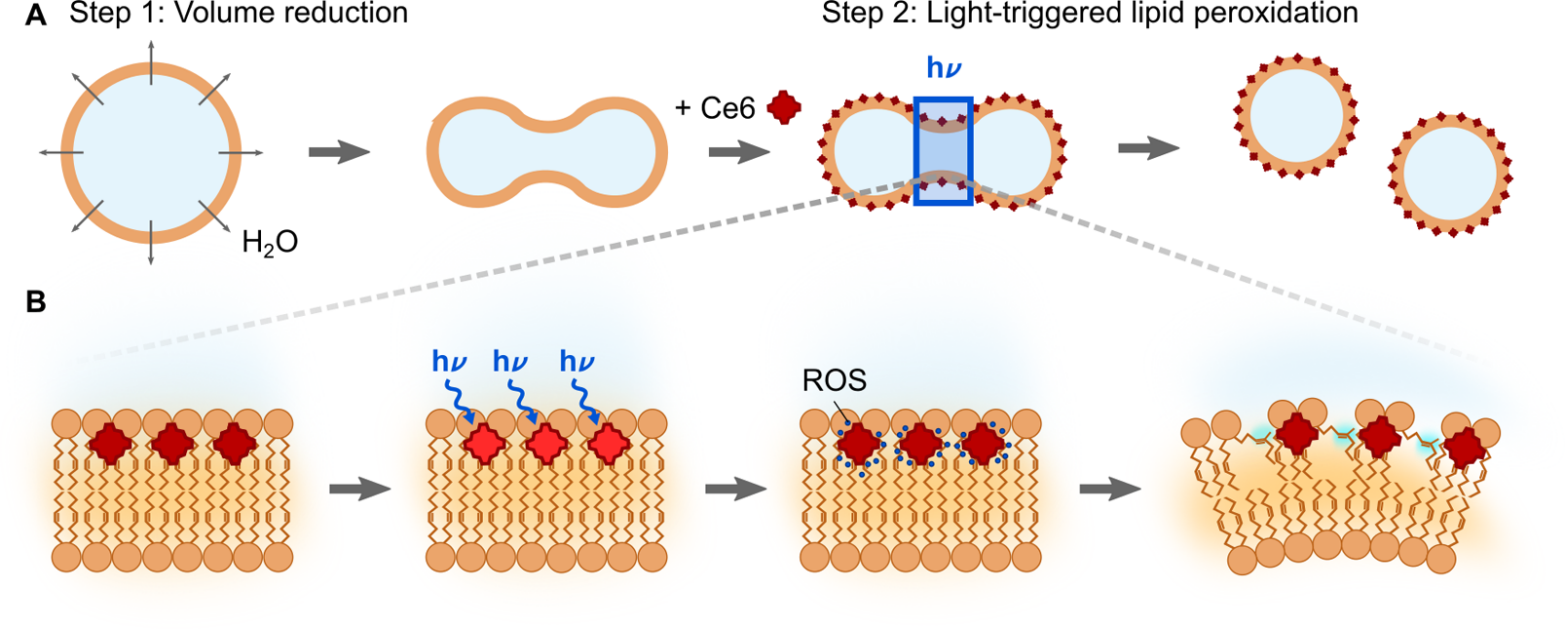
Schematic of the proposed two-step mechanism for the light-triggered division of GUVs. (A) In the first step, GUVs are osmotically deflated to provide sufficient excess membrane area for division. In a second step, seconds of illumination lead to local lipid peroxidation of the outer membrane leaflet in the presence of the photosensitizer Ce6. This increases the spontaneous curvature and thus enables neck fission. (B) Mechanism of Ce6-mediated lipid peroxidation. When added to GUVs from the outside, Ce6 self-assembles into the outer leaflet of the lipid bilayer. Local illumination with light of the wavelength 405 nm triggers the generation of reactive oxygen species (ROS) in close proximity to the lipid tails. The ROS cause lipid peroxidation in the outer leaflet and hence an asymmetric area increase.

Before realizing GUV division, we first verify that Ce6-mediated lipid peroxidation occurs in our GUV system and demonstrate Ce6-mediated reversible cargo loading as a valuable second functionality for bottom-up synthetic biology. For this purpose, we use GUVs at iso-osmotic conditions and trigger the oxidation of the lipids either in bulk by placing the sample under a UV lamp or by illuminating single GUVs with the 405 nm laser of a confocal microscope. To maximize the effect of Ce6 with minimal light exposure, we choose a Ce6 concentration of 100 μM which is close to the maximal solubility (∼235 μM^28^). Photoinduced lipid peroxidation has been shown to increase the membrane permeability for sugars on the time scale of minutes.^25^ Here, we test if this observation can be extended to charged compounds, which would be useful to implement a loading step for synthetic cells. Therefore, we add the positively charged membrane-impermeable dye Alexa647-NHS ester to GUVs in the presence of Ce6. After mixing, the dye remains in the outer aqueous phase surrounding the GUV (Figure 2A, left image). This confirms that despite the fact that the autofluorescent Ce6 molecules are colocalizing with the lipid bilayer (Supporting Figure S1), they do not permeabilize the membrane. However, during pulsed illumination of the entire GUV with a 405 nm laser, the fluorophore permeates into the GUV (Figure 2A, right image). In the absence of Ce6, no influx can be detected after illumination (Figure 2B). By analyzing the fluorescence intensity inside the GUV over time with confocal imaging, we detect dye influx starting after 5 min of pulsed illumination for GUVs in the presence of Ce6 (Figure 2C). The influx proceeds along the concentration gradient with a time constant of τ = 3.35 min until the inner and outer dye concentrations are equilibrated. We further demonstrate the high spatiotemporal control over the permeabilization by locally illuminating a single GUV. The selected GUV shows dye influx whereas the surrounding ones remain unaffected (Supporting Figure S2). Beyond fluorophores, the Ce6-mediated permeabilization provides the possibility to load larger and highly charged polymers into the GUVs. Of particular interest, we were able to load single-stranded DNA into GUVs (Supporting Figure S3). Concurrently to the membrane permeabilization, we observe a shrinkage of GUVs to about a quarter of the initial volume (corresponding to a 37% reduction in diameter) when the entire GUV is subject to pulsed illumination over the course of 20 min (Figure 2D, Supporting Figure S4). This confirms that lipid peroxidation is taking place, which, after an initial area increase in the outer bilayer leaflet, could lead to lipid desorption from the membrane. This would be a possible explanation for the effective volume decrease (see Supporting Note 1).^25^ With mass spectrometry analysis, we confirmed the occurrence of oxidized lipids. Note that we also confirmed the presence of unsaturated DOPC even after extended illumination times (Supporting Figure S5).

**Figure 2:**
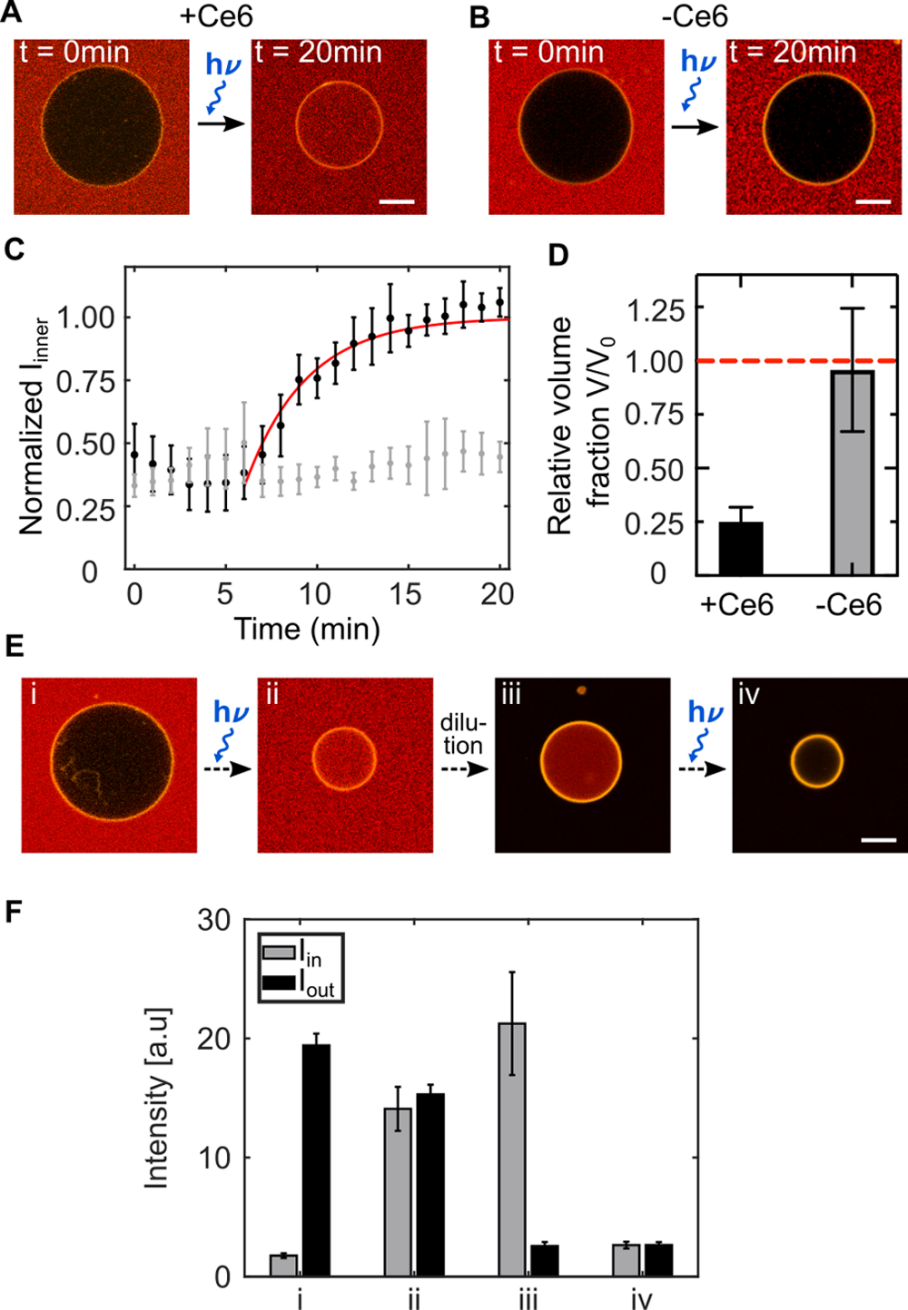
Light-triggered cargo loading of GUVs mediated by Ce6. (A,B) Confocal images of GUVs (DOPC lipids, labeled with 1% Rhodamine-PE, λ_*ex*_ = 561 nm, orange) immersed in a solution of Alexa647-NHS ester (λ_*ex*_ = 633 nm, red) in the presence (A) and absence of Ce6 (B). GUVs were illuminated every 10 s for 1 s with a 405 nm laser leading to dye influx and volume reduction only in the presence of Ce6. Scale bars: 10 μm. (C) Normalized intensity of Alexa647-NHS ester inside GUVs over time in the presence (black) and in the absence (gray) of Ce6. After 6 min, the dye permeates into the GUVs along the concentration gradient. The dye influx was fitted with 1 – *c*_0_ exp((*t* – *t*_0_)/τ) with the time constant τ = 3.35 min (red curve). (D) Relative volume fraction *V*/*V*_0_ of GUVs in the presence and absence of Ce6. Over the course of 20 min of periodical illumination with a 405 nm laser, GUVs in the presence of Ce6 shrink to about a quarter of their initial volume *V*_0_, whereas the volume remains constant during illumination in the absence of Ce6. Error bars correspond to the standard deviation for *n* = 5 (C) and *n* = 10 (D) different GUVs. (E) Confocal images of GUVs (labeled with Rhodamine-PE, λ_*ex*_ = 561 nm, orange) immersed in a solution of Alexa488 carboxylic acid succinimidyl ester (λ_*ex*_ = 488 nm, red) in the presence of Ce6. GUVs can be loaded and unloaded sequentially upon illumination for 15 min with a UV lamp. Scale bar: 10 μm. (F) Mean intensity inside (*I*_in_, gray) and outside (*I*_out_, black) GUVs during different stages of the sequential loading and unloading process (i–iv, as indicated in E). The error bars correspond to the standard deviation of the mean (*n* = 3–6).

Importantly, this provides us with a strategy to load and unload a cargo from a selected GUV in a controlled manner as demonstrated in Figure 2E. Upon UV illumination in bulk with a UV lamp for 15 min in the presence of Ce6, an otherwise membrane impermeable cargo permeates into the GUV along its concentration gradient (ii). After purification of the GUV by dilution, the cargo remains enclosed inside the GUV, proving that the transient pores in the GUV membrane close after illumination and an intact lipid bilayer reforms (iii). Since controlled release is equally important as controlled encapsulation, we next unload the cargo using the same mechanism. We again illuminate the GUVs for 15 min to induce pore formation. Since the GUV volume is small compared to the reservoir, we obtain virtually complete cargo export (iv). To quantify the loading and unloading we analyzed the dye intensity inside and outside the GUVs at all four stages (Figure 2F). This clearly demonstrates stable encapsulation (iii) and efficient release (iv). Importantly, we verified with quantitative polymerase chain reaction (qPCR) experiments that biocomponents remain functional after 15 min of continuous UV illumination as used here (Supporting Figure S6). The fact that pore formation can be induced multiple times demonstrates that only a fraction of the lipids is oxidized even after extended illumination times. This showcases that the photosensitizer Ce6 offers full spatiotemporal control to sequentially load and unload GUVs with cargo in a reversible manner.

Next, we set out to employ the Ce6-mediated lipid peroxidation for the proposed localized GUV division. At constant membrane area, division requires a volume decrease due to the increased surface-to-volume ratio of the two daughter GUVs. Considering the vesicle geometry, we calculate that a volume reduction by a factor of √2 provides the required membrane area for the GUVs to form two equally sized buds.^16^ To ensure sufficient excess membrane area, we deflate the GUVs to a volume ratio of ν = 1.66 by slow stepwise addition of a higher osmolarity sucrose solution. In this state, the GUVs can take on several prominent shapes, such as prolate, oblate, stomatocyte and discocyte and for nonvanishing spontaneous curvature the formation of buds connected with a tight neck.^14^ The GUVs can occasionally undergo transitions between these states.^15^ From previous theoretical work it is known that increasing the spontaneous curvature of prolate-shaped GUVs leads to a state where two GUVs are connected with a tight neck.^14^ Here, we achieve the increase in spontaneous curvature by taking advantage of the initial area increase associated with the first reaction step of Ce6-mediated lipid peroxidation of prolate-shaped GUVs (Figure 3A, Supporting Video 1). The transition from prolate to two vesicles connected with a tight neck happens within seconds of 405 nm laser illumination, confirming the fast dynamics of the peroxidation process that precedes pore formation (Figure 3A, i-iii). Subsequent illumination of the neck region again causes a local increase of spontaneous curvature which mediates complete fission of the neck regime (Figure 3A, (iv). Over time the divided daughter vesicles diffuse apart, confirming their complete division. Due to independent diffusion in the *z*-direction, the GUVs do not remain in the same confocal plane, which is why we provide brightfield images after division. Due to the broad absorbance spectrum (Supporting Figure S7) and the autofluorescence of Ce6 (Supporting Figure S8), it is not possible to verify successful fission by fluorescence recovery after photobleaching (FRAP) experiments which study the recovery of the lipid fluorescence in the daughter vesicles. However, after the division of Atto488-NHS-loaded GUVs we observe unequal but constant fluorescence intensity in both vesicles. This confirms complete neck scission and the absence of lipid tubes which would allow for an equilibration of the fluorescence signal (see Supporting Figure S9). Furthermore, z-stacks after division do not show any lipid tubes that connect the GUVs. From the kymograph in Figure 3B, one can appreciate the continuous constriction of the neck region, which eventually leads to a closed neck structure after only 15 s of illumination. Note that the oxidized lipids are likely excluded from the lipid bilayer (see Supporting Note 1),^25^ such that the spontaneous curvature vanishes and the starting conditions for consecutive division cycles can be restored. From mass spectrometry experiments we know that sufficient intact DOPC lipids remain (Supporting Figure S5).

**Figure 3:**
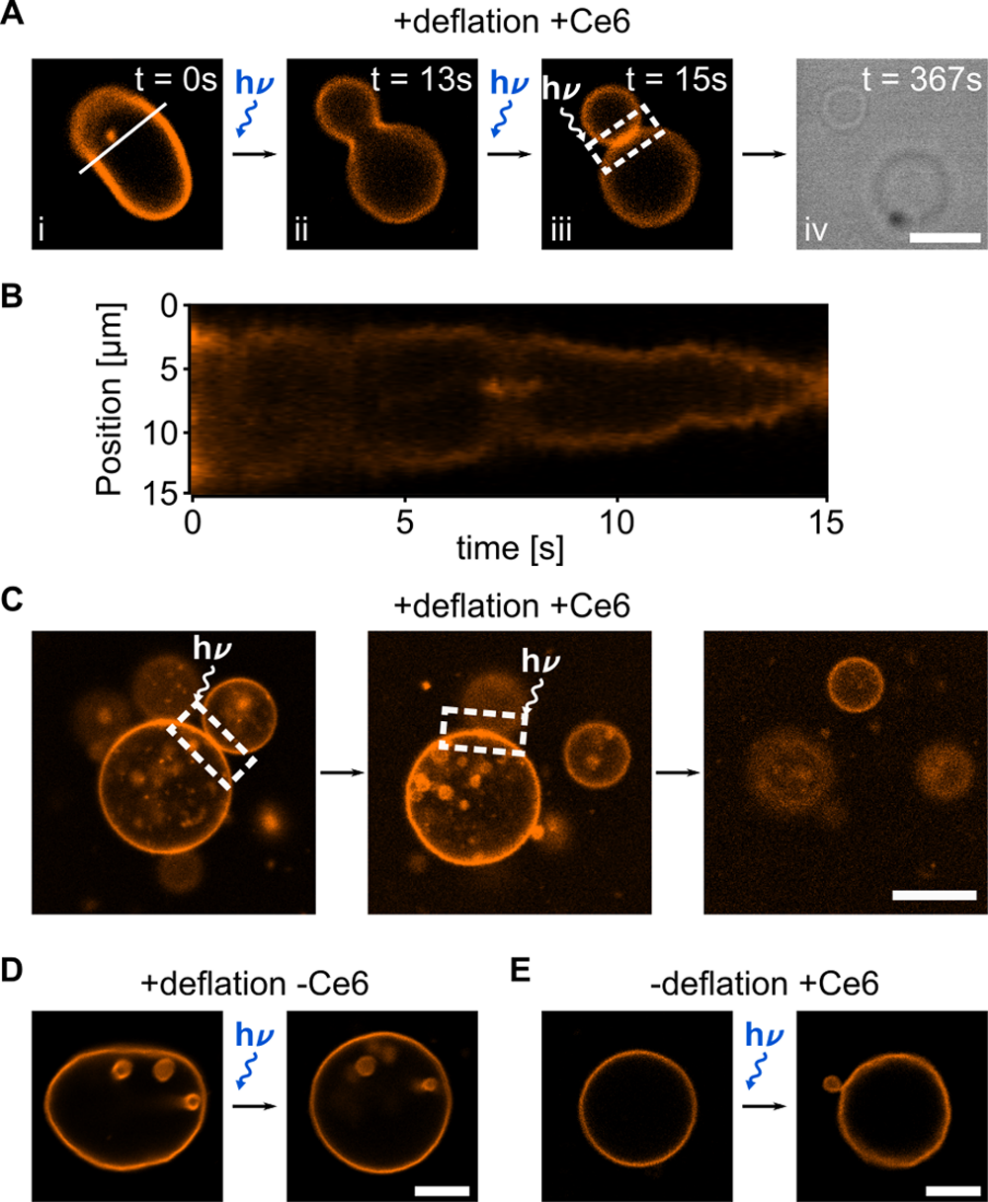
Local GUV division by light-triggered Ce6-mediated lipid peroxidation. (A) Confocal images of a GUV (DOPC lipids, labeled with 1% Rhodamine-PE, λ_*ex*_ = 561 nm) at different time points during and after illumination with a 405 nm laser (total illumination time 100 s). (B) Kymograph of the division process shown in (A) plotting the intensity profile along the white line. (C) Confocal images of a deflated GUV forming multiple buds. Consecutive illumination of the neck regions (indicated by white dashed boxes) with a 405 nm laser leads to consecutive fission of the buds. (D) Deflation alone leads to bud formation to store excess membrane area but no fission was observed (*n* = 150 GUVs, illumination time ≥1 min with a 405 nm laser). (E) A GUV under iso-osmotic condition in the presence of Ce6 shows formation of a small bud upon 405 nm laser illumination due to an increase of the membrane area and spontaneous curvature, however, division into equally sized compartments does not occur (*n* = 150 GUVs, illumination time ≥1 min). Scale bars: 10 μm.

Osmotic deflation of GUVs to a volume ratio of ν = 1.66 provides sufficient excess membrane area to allow for the formation of multiple buds as exemplified in the confocal image in Figure 3C. In this state, we can demonstrate the level of control over the division process by inducing fission of one bud after the other. This is achieved by consecutive 405 nm laser illumination of the neck regions for about 30 s each. In this way, we obtain three separate GUVs from a single GUV, which highlights the good reproducibility of the division mechanism and mimics nonbinary fission of natural cells.^29^ Division after illumination of the neck region of GUVs happens reproducibly (see Supporting Note 2). 10–50 s after the start of the illumination, about 50% of the GUVs suddenly start to diffuse up to 80 μm apart, which indicates the complete division into two daughter compartments. In total, we observed *n* = 21 fission events out of 200 vesicles that were subjected to the process. For 6 of those, we observed the entire constriction process leading to the formation of a dumbbell shape with the subsequent fission of the daughter vesicles (see Supporting Figure S10 for additional examples and Supporting Note 2 for a statistical analysis).

In control experiments we show that illumination of deflated GUVs alone, in the absence of Ce6, leads to the described membrane fluctuations and shape transformations. However, fission was never observed even after illumination of 1–3 min for *n* = 150 GUVs–neither for illumination of the whole vesicle (Figure 3D) nor when the neck regime between two buds was illuminated (Supporting Figure S11). Similarly, GUV division upon illumination could not be obtained in the presence of Ce6 without the initial deflation step in control experiments with *n* = 150 GUVs. The Ce6-mediated increase in spontaneous curvature leads to GUV morphology changes such as bud formation, however, we never observed division (Figure 3E). Taken together, this confirms the proposed two-step division mechanism, which requires both an increase in the surface-to-volume ratio and an increase in spontaneous curvature mediated by Ce6-triggered lipid peroxidation upon illumination. In Supporting Table 1 we provide an overview of the statistics of the division process for the experiments and the controls, and in Supporting Note 2 we confirm the statistical significance of our observations.

We have thus demonstrated that two completely distinct, yet highly complementary processes, namely sequential cargo loading and unloading as well as GUV division, can be achieved with full spatiotemporal control by simple addition of Ce6 to a GUV solution. It is important to note that, even though both processes are based on lipid peroxidation, they can be triggered independently of one another due to the different spatiotemporal requirements. While an initial spontaneous curvature increase which enables GUV division happens within seconds of illumination, pore formation and cargo import or export require illumination of the entire GUV for tens of minutes.

Importantly, a suitable division mechanism for bottom-up synthetic biology should provide the possibility to achieve the division of GUVs loaded with an information-storing substrate. Thus, we demonstrate the division of DNA-containing GUVs. We load the GUVs with fluorescently labeled single-stranded DNA during the electroformation process. This avoids bleaching of the lipids and the DNA due to UV illumination prior to the division process. Moreover, we noticed that after extended periods of UV illumination, the Ce6 seems to distribute evenly in both membrane leaflets likely due to pore formation. Therefore, a resupply of Ce6 after the initial loading step would be necessary. After verifying the successful and stable encapsulation of the DNA inside the GUV lumen (Supporting Figure S12), the GUVs are deflated. Directly after addition of Ce6, a target GUV is selected and illuminated. Figure 4A depicts an exemplary time series of the division process of such a DNA-containing GUV. Within seconds of illumination, morphology changes can be observed from a prolate to a dumbbell shape until neck fission occurs after 53 s (Supporting Video 2). The confocal image in Figure 4B, taken after 350 s, proves the complete fission. The then spherical daughter GUVs diffuse apart. Notably, from the fluorescence intensity distribution, we can infer that the daughter GUVs stably encapsulate equal amounts of DNA after division (see Supporting Figure S13). Note that the same brightness and contrast settings were applied for all images, the decrease in intensity is a result of enhanced bleaching due to 405 nm illumination.

**Figure 4:**
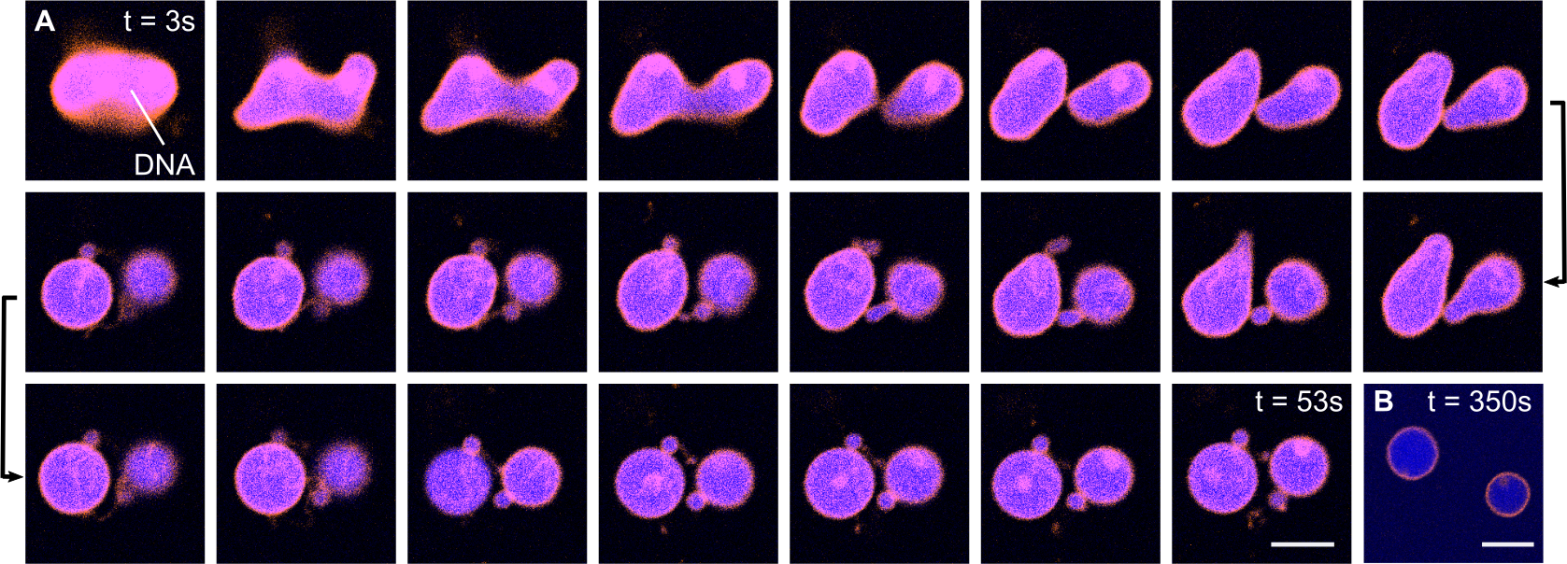
Ce6-mediated division of DNA-containing GUVs. (A) Confocal time series of a GUV (orange, DOPC lipids, labeled with Rhodamine-PE, λ_*ex*_ = 561 nm) containing 5 μM single-stranded DNA (blue, labeled with Atto488, λ_*ex*_ = 488 nm) undergoing the Ce6-mediated division process during illumination with a 405 nm laser for 53 s. The GUV morphology changes from a prolate to a dumbbell shape until complete neck fission occurs after 53 s of illumination. The time steps between the images are 2.3 s. (B) After division, the daughter GUVs diffuse apart. All images are shown with identical contrast and brightness settings, the reduction in intensity is due to bleaching. Scale bars: 10 μm.

The combination of synthetic cell division with information propagation is a fundamental milestone for bottom-up synthetic biology and one of the most exciting hurdles toward the creation of artificial life. The division of DNA-containing GUVs as achieved here delineates a crucial first step for coupling compartment division with an informational substance. By simple addition of the photosensitizer Ce6 to the GUV solution, we divide osmotically deflated GUVs locally within seconds of illumination. We select single GUVs in an ensemble and initiate their division process with full spatiotemporal control. Importantly, the mechanism is suitable for sustained division, as the starting conditions are, in principle, restored. Although only a small fraction of the lipids is oxidized in each cycle, sustained division will eventually require a mechanism for lipid regeneration. The addition of Ce6 can also be exploited for a completely different purpose, namely sequential loading and unloading of GUVs. Although pore formation also relies on lipid peroxidation, it can be triggered independently of division, since the latter requires osmotic deflation in combination with an order of magnitude shorter illumination times. Beyond synthetic biology, controlled cargo encapsulation and release is of great importance for liposome-based drug delivery systems. We thus derived an easy applicable multipurpose tool to manipulate GUVs on the single-compartment level with light, which can directly be implemented by laboratories around the world. Most importantly, the localized division of DNA-containing GUVs opens up the possibility to combine cell division with other synthetic cell modules—ultimately applicable for the directed evolution of synthetic cells.

## Supporting Information Available

Materials and methods; Figures S1–S13 of Ce6 incorporated into the lipid bilayer, localized increase of GUV permeability, DNA encapsulation, volume decrease, MS, UV, and absorbance spectra, autofluorescence, proof of vesicle fission, examples of GUVs undergoing light-triggered division, fission of deflated GUVs, DNA after encapsulation; Notes 1 and 2 of volume shrinkage and division mechanism (PDF)
Video 1: Ce6-mediated division of GUVs in comparison with respective controls (AVI)
Video 2: Ce6-mediated division of DNA-containing GUVs (AVI)

## Supplementary Material

nl1c00822_si_001.pdf

nl1c00822_si_002.avi

nl1c00822_si_003.avi
