# Supplementary material for: Light-Triggered Cargo Loading and Division of DNA-Containing Giant Unilamellar Lipid Vesicles
Source: Nano Lett. 2021 Jul 12;21(14):5952–7. doi: 10.1021/acs.nanolett.1c00822 (PMC8323123; doi:10.1021/acs.nanolett.1c00822)
Supplement: Supplementary file 1 — nl1c00822_si_001.pdf [file nl1c00822_si_001.pdf]

# Supporting Information: Light-Triggered Cargo Loading and Division of DNA-Containing Giant Unilamellar Lipid Vesicles

Yannik Dreher,[†,‡,§] Kevin Jahnke,[*,†,‡,§] Martin Schröter,[¶] and Kerstin Göpfrich[*,†]

†*Max Planck Institute for Medical Research, Biophysical Engineering Group, Jahnstraße 29, 69120 Heidelberg, Germany*

‡*Department of Physics and Astronomy, Heidelberg University, 69120 Heidelberg, Germany*

¶*Max Planck Institute for Medical Research, Department of Cellular Biophysics, Jahnstraße 29, 69120 Heidelberg, Germany*

§*These authors contributed equally.*

E-mail: kevin.jahnke@mr.mpg.de; kerstin.goepfrich@mr.mpg.de

# Contents

**1 Experimental Methods** **4**

1.1 GUV formation . . . . . . . . . . . . . . . . . . . . . . . . . . . . . . 4

1.2 Confocal imaging . . . . . . . . . . . . . . . . . . . . . . . . . . . . . 4

1.3 Determination of the osmolality . . . . . . . . . . . . . . . . . . . . . 5

1.4 Mass spectrometry analysis . . . . . . . . . . . . . . . . . . . . . . . . 5

1.5 Ce6-mediated dye influx into GUVs . . . . . . . . . . . . . . . . . . . 6

1.6 Cargo loading and unloading from GUVs . . . . . . . . . . . . . . . . 6

1.7 Quantitative polymerase chain reaction (qPCR) measurements: . . . . . . . 6

1.8 Ce6-mediated local division . . . . . . . . . . . . . . . . . . . . . . . 7

1.9 Ce6-mediated local division of DNA-containing GUVs . . . . . . . . . . . . 8

**2 Supporting Figures** **9**

2.1 Figure S1: Ce6 is incorporated into the lipid bilayer of GUVs . . . . . . . . . 9

2.2 Figure S2: Localized increase of GUV permeability can be controlled with light 10

2.3 Figure S3: DNA encapsulation by Ce6-mediated permeability increase of GUVs 11

2.4 Figure S4: Volume decrease of GUVs in presence of Ce6 after light illumination 12

2.5 Figure S5: Mass spectrometry confirms Ce6-mediated lipid peroxidation . . 13

2.6 Figure S6: UV illumination dose as used for vesicle loading does not affect the functionality of bio-molecules . . . . . . . . . . . . . . . . . . . . . . . 15

2.7 Figure S7: Absorbance spectrum of Ce6 . . . . . . . . . . . . . . . . . . . 16

2.8 Figure S8: Autofluorescence of Ce6 prevents bleaching after division as proof for full fission . . . . . . . . . . . . . . . . . . . . . . . . . . . . . 17

2.9 Figure S9: Proof of vesicle fission . . . . . . . . . . . . . . . . . . . . . 19

2.10 Figure S10: Examples of GUVs undergoing light-triggered division in the presence of Ce6 . . . . . . . . . . . . . . . . . . . . . . . . . . . . . 20

2.11 Figure S11: 405 nm illumination alone is not sufficient to achieve fission of deflated GUVs . . . . . 21
2.12 Figure S12: DNA remains intact after encapsulation into GUVs . . . . . 22
2.13 Figure S13: DNA remains encapsulated in the daughter vesicles after Ce6-mediated division. . . . . . 23

**3 Supporting Notes 24**

3.1 Supporting Note 1: Ce6-mediated volume shrinkage . . . . . 24
3.2 Supporting Note 2: Statistics and reproducibility of the division mechanism . 25

**4 Supporting Videos 26**

4.1 Supporting Video 1: Ce6-mediated division of GUVs . . . . . 26
4.2 Supporting Video 2: Ce6-mediated division of DNA-containing GUVs . . . . 26

**References 27**

# 1 Experimental Methods

## 1.1 GUV formation

1,2-dioleoyl-sn-glycero-3-phosphocholine (18:1 DOPC) and 1,2-dioleoyl-sn-glycero-3-phospho ethanolamine-N-(lissamine rhodamine B sulfonyl) (ammonium salt) (18:1 Rhodamine PE) were purchased from Avanti Polar Lipids, Inc., and stored in $CHCl_3$ at −20 °C. Giant unilamellar vesicles (GUVs) were produced via the electroformation method [1] using a VesiclePrepPro device (Nanion Technologies GmbH). 40 µL of 1 mM lipid mix (containing 99 % DOPC and 1 % Rhodamine PE) in $CHCl_3$ were homogeneously spread on the conductive side of an indium tin oxide (ITO) coated glass slide (Visiontek Systems Ltd) using a cover slide. The lipid-coated ITO slide was subsequently placed under vacuum for at least 10 min to achieve complete evaporation of the $CHCl_3$. A rubber ring with a diameter of 18 mm was placed on the lipid-coated ITO slide. The ring was filled with 275 µL of 60 mM sucrose solution, which was preheated to 60 °C, before creating a sealed chamber by placing a second ITO slide on top. The assembled electroformation chamber was placed into the VesiclePrepPro and connected to the electrodes. An AC field (3 V, 5 Hz) was applied across the ITO slides for 126 min while heating the solution to 37 °C. GUVs were collected immediately after formation and stored at 4 °C for up to 7 days.

## 1.2 Confocal imaging

For confocal microscopy, the GUV-containing solution was sealed in a BSA-coated observation chamber consisting of two cover slides spaced by a sticky tape (tesa) and imaged using a confocal laser scanning microscope LSM 880 or LSM 900 (Carl Zeiss AG). The pinhole aperture was set to one Airy Unit and experiments were performed at room temperature. The images were acquired using a 20x objective (Plan-Apochromat 20x/0.8 M27, Carl Zeiss AG). Images were analyzed and processed with ImageJ (NIH, brightness and contrast adjusted).

Data was analyzed and plotted using Matlab (2019) and Graphpad Prism (2019).

### 1.3 Determination of the osmolality

The osmolality of all solutions was measured with an Osmomat 030 (Gonotec GmbH). Before use, the osmometer was calibrated with calibration solutions of 0 and 300 mOsm/kg (Gonotec GmbH). Each measurement was carried out with a sample volume of 50 µL. Note that here the osmolality can be used as a good approximation for the osmolarity.[2]

### 1.4 Mass spectrometry analysis

The effects of irradiating SUVs with UV light, in presence and in absence of Ce6, were studied by mass spectroscopy. For this purpose, the Bruker maXis ETD II system was used. Four samples were prepared: (I) 100 µM SUVs in deionized water, (II) 100 µM SUVs in deionized water, which were irradiated for 5 min with UV light, (III) 100 µM SUVs and 100 µM Ce6 in deionized water, and (IV) 100 µM SUVs and 100 µM Ce6 in deionized water, which were irradiated for 5 min with UV light. For the analysis, two different buffers were used:

Buffer A: deionized water + 0.1 % formic acid

Buffer B: acetonitrile + 0.1 % formic acid

The analysis took place via a flow injection analysis on a device Shimadzu Nexera X2 system (450 µL $min^{-1}$ 70% Buffer B). Each sample was diluted 1:10000 in 70 % Buffer B and 10 µL of the samples were injected. The mass spectra were recorded in positive mode in a mass range from 250 to 1500 m/z (Source parameters: End plate offset: 500 V, Capilary: 3800 V, Nebulizer: 45.0 psi, dry gas: 10 L $min^{-1}$, dry temperature: 250 °C). Echo-peak internal standard calibration based on NH4form clusters. Bruker Compass otofControl Version 5.2 (Build 0.8) and Bruker Compass HyStar 5.1.8.1 were used as control software and Bruker Compass DataAnalysis 5.3 (Build 342.363.6049)(64-bit) for analysis.

## 1.5 Ce6-mediated dye influx into GUVs

GUVs were mixed with 100 µM Ce6 (Cayman Chemicals) and 50 µM Alexa647-NHS ester as membrane impermeable dye and permeability indicator. GUVs were imaged with confocal microscopy every 10 s for 27 min. During each acquisition cycle, the GUV was subjected to 1 s of illumination with 405 nm at 70 % of the intensity of a 5 mW laser diode. To analyze the dye influx over time, a spherical region of interest with a diameter of 50 pixels inside the GUV was chosen and the intensity was analyzed with ImageJ. The intensities were normalized to the intensity outside the GUV, which was determined from a circle with a diameter of 100 pixels.

## 1.6 Cargo loading and unloading from GUVs

GUVs were mixed with 50 µM of the membrane impermeable dye Alexa488 carboxylic acid succinimidyl ester (Sigma-Aldrich) and 100 µM Ce6. GUVs were then placed under a UV lamp (Hamamatsu) equipped with a 365 nm filter for 15 min. Afterwards, they were imaged and subsequently diluted at a volumetric ratio of 1:10 with a 60 mM sucrose solution. To subsequently release the dye from the GUVs, GUVs were placed again under the UV lamp for 15 min and imaged afterwards.

## 1.7 Quantitative polymerase chain reaction (qPCR) measurements:

Fast SYBR$^{TM}$ Green Master Mix (catalog number: 4385610) was purchased from Thermo Fisher Scientific. Two identical samples of 500 µl were prepared containing 1x SYBR$^{TM}$ Green Master Mix solution, 300 nM primers (primer1 sequence: 5'- CCC ATG TTC TGG CTG CAC TCC AGG CTG GGA ATC GTG TGA AGG TTT CTC TCG AGC CAA C -3' , primer 2 sequence: 5'- CGA TAT CAC TAG TGC TAC CAC CAC TTC CTC CTC CTC

CAC TTC CTC -3', from Integrated DNA Technologies, purification: HPLC) and 1 ng $\mu l^{-1}$ DNA template (13 kbp). One sample was illuminated with a UV lamp in a distance of about 3 cm (Hamamatsu, power: 20 mW $cm^{-2}$ at 5 cm distance) for 15 min before initiation of the PCR cycles. Subsequently both samples were divided into ten aliquots of 50 µl. To run the polymerase activation step and the subsequent PCR cycles a C1000 Touch™ Thermal Cycler from Bio-Rad was used. All aliquots were heated up to 95 °C for 10 min as an initial activation step for the polymerase. One aliquot of each sample was removed from the thermal cycler (n = 0 cycles) and stored at 4 °C. Next, eleven PCR cycles were run. Each cycle consisted of 10 sec at 95 °C and 1 min at 60 °C. After each run of this protocol one aliquot of each sample was removed from the thermal cycler and stored at 4 °C. After all cycles were collected, their emission intensity spectra were analyzed from 515 −600 nm using a Spark plate reader from TECAN (excitation wavelength: 476 nm, excitation bandwidth: 20 nm).

## 1.8 Ce6-mediated local division

GUVs with an initial sucrose concentration of 60 mM were diluted with a 120 mM sucrose solution to a final concentration of 100 mM sucrose to induce the osmotic deflation of the vesicles. The dilution was performed stepwise (10 steps) over the course of 1 h in order to avoid tubulation of the vesicles due to the osmotic shock.[2] Subsequently, the diluted vesicle solution was mixed at a volumetric ratio of 1:1 with a solution containing 200 µM Ce6 dissolved in 100 mM sucrose. The GUVs were imaged with confocal microscopy. Lipid peroxidation and thus division in the presence of Ce6 was triggered by illumination with the 405 nm diode laser at 60 % laser intensity (5 mW) for up to 100 s. Control experiments were performed with deflated GUVs without Ce6 and by mixing undeflated GUVs 1:1 with an iso-osmolar solution containing 200 µM Ce6 and 60 mM sucrose. Control samples were illuminated for up to 3 min with the 405 nm diode laser at 60 % laser intensity.

## 1.9 Ce6-mediated local division of DNA-containing GUVs

DNA-containing GUVs were prepared using the electroformation method as before by adding 5 µM Atto488-labeled single-stranded DNA (ssDNA, 5'-Atto488-AAAAAAAAA AAAAAAAAAAA-3', from Biomers) to a 60 mM sucrose solution. Subsequently, GUVs were deflated (5 steps) to an osmolarity ratio of 1.66 using a 120 mM sucrose solution. Afterwards, DNA-containing GUVs were mixed with Ce6 at a final concentration of 100 µM. Lipid peroxidation in the presence of Ce6 was triggered by illumination with the 405 nm diode laser at 70 % laser intensity (5 mW) for up to 70 s.

# 2 Supporting Figures

## 2.1 Figure S1: Ce6 is incorporated into the lipid bilayer of GUVs

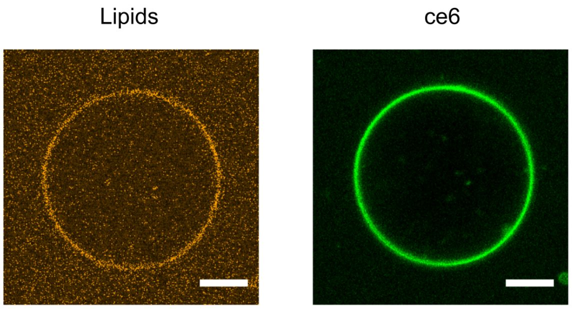


Figure 1: Ce6 is incorporated into the lipid bilayer of GUVs. Confocal image of a GUV (DOPC, labeled with 1 % Rhodamine-PE, $\lambda_{ex} = 561$ nm, orange) in 60 mM sucrose mixed with 100 µM Ce6 ($\lambda_{ex} = 405$ nm, green). The emission of the auto-fluorescent Ce6 was detected with a detection window from 640 nm-700 nm. Ce6 clearly colocalizes with the GUV membrane and leads to quenching of Rhodamine-PE. Scale bars: 10 µm.

## 2.2 Figure S2: Localized increase of GUV permeability can be controlled with light

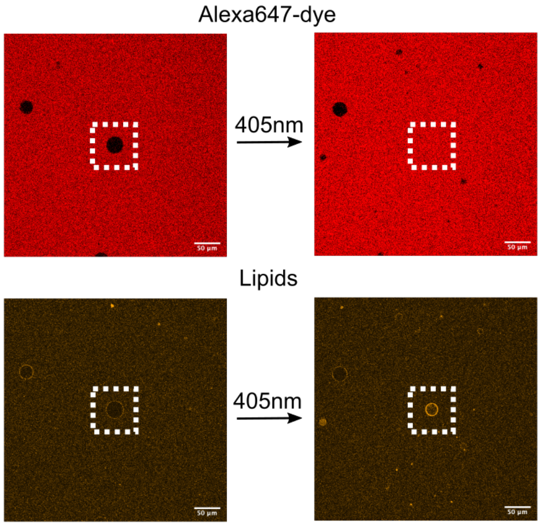


Figure 2: Localized increase of GUV permeability can be controlled with light. Confocal overview image of GUVs ($\lambda_{ex} = 561\,\text{nm}$) mixed with $50\,\mu\text{M}$ of the membrane impermeable dye Alexa647-NHS ester ($\lambda_{ex} = 633\,\text{nm}$) and $100\,\mu\text{M}$ Ce6. The GUV in the centre of the frame (highlighted by the white dashed box) was locally illuminated with a $405\,\text{nm}$ laser diode for $27\,\text{min}$ leading to dye influx into the illuminated GUV. Surrounding GUVs remained impermeable. This showcases the high spatio-temporal control to permeabilize GUVs locally. Scale bars: $50\,\mu\text{m}$.

## 2.3 Figure S3: DNA encapsulation by Ce6-mediated permeability increase of GUVs

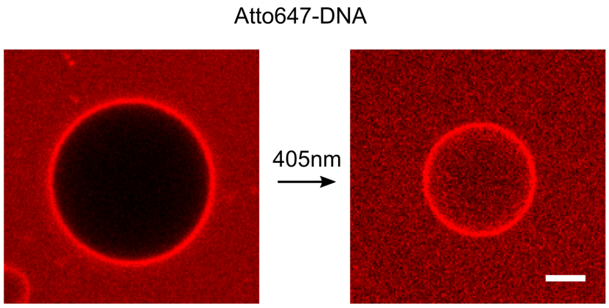


Figure 3: DNA encapsulation by Ce6-mediated permeability increase of GUVs. Confocal image a GUV mixed with 5 µM Atto647-labeled single-stranded DNA ($\lambda_{ex} = 633$ nm, sequence: 5' Atto647N-TTC TCT TCT CGT TTG CTC TTC TCT TGT GTG GTA TTG TCT AAG AGA AGAG 3', HPLC purified, from Biomers)) and 100 µM Ce6. The GUV was illuminated with a 405 nm laser diode for 35 min and became permeable to the DNA and shrunk in volume. Scale bar: 10 µm.

## 2.4 Figure S4: Volume decrease of GUVs in presence of Ce6 after light illumination

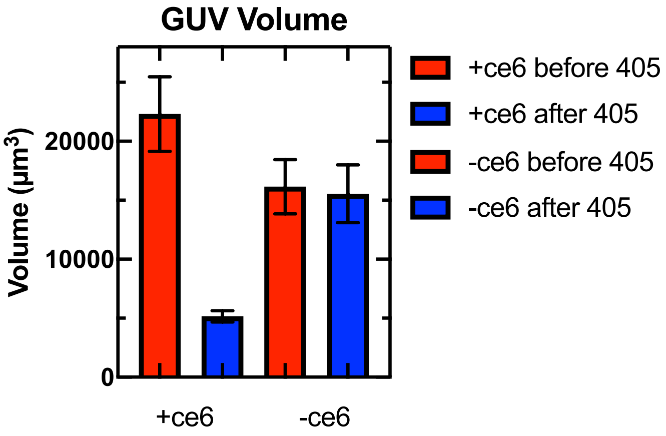


Figure 4: Volume decrease of GUVs in presence of Ce6 after light illumination. GUV volume in presence and absence of Ce6 before (red) and after (blue) illumination with 405 nm for 27 min. In presence of Ce6 (100 µM) the GUV volume decreases to about a quarter after 405 nm illumination, whereas the GUV volume remains constant without Ce6. Note that after the end of the illumination period, the GUV volume remains constant in all cases. Error bars correspond to the standard deviation of n=10 GUVs.

## 2.5 Figure S5: Mass spectrometry confirms Ce6-mediated lipid peroxidation

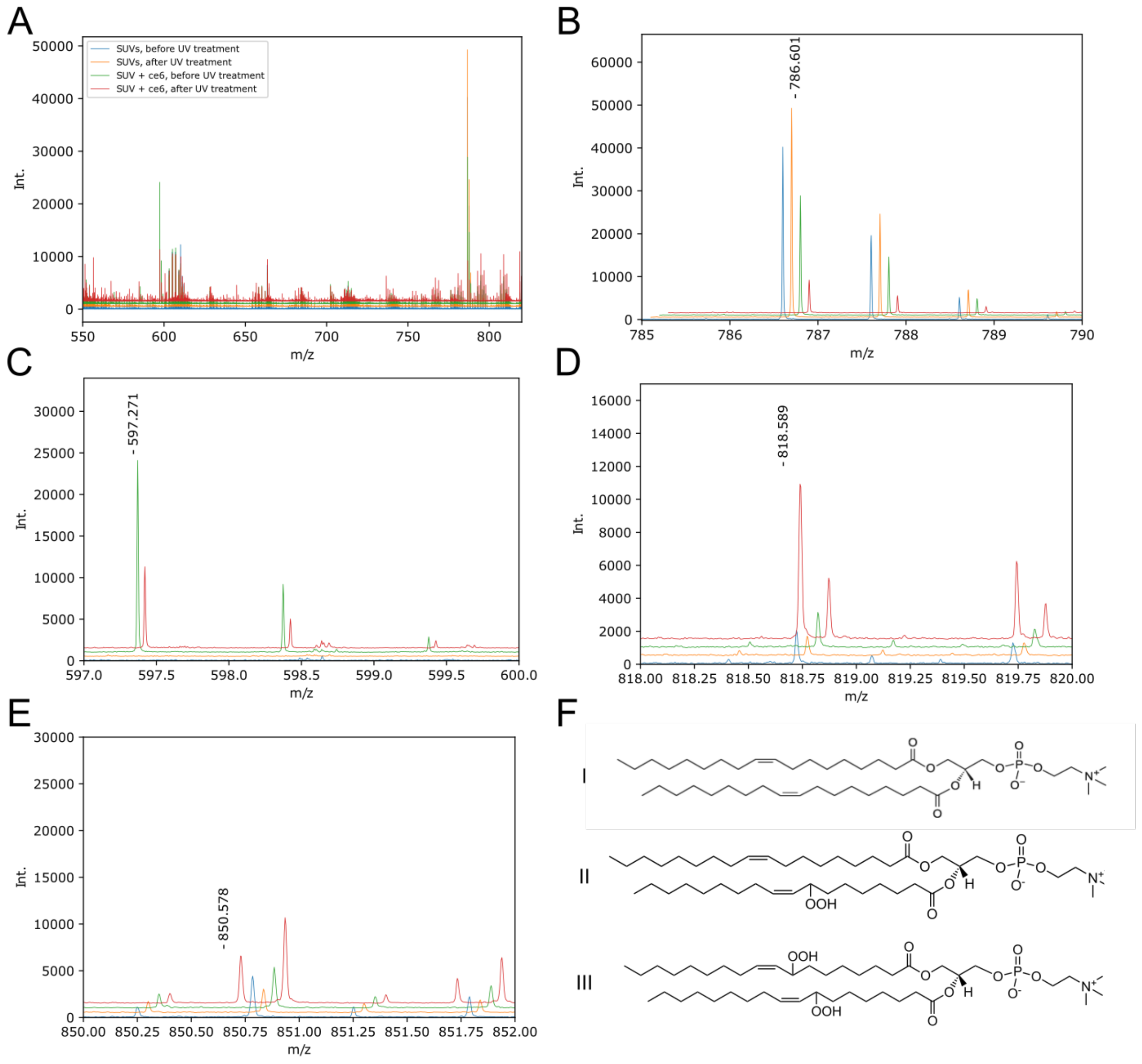


Figure 5: Mass spectrometry confirms Ce6-mediated lipid peroxidation upon illumination for 5 minutes. (continued on the following page)

Figure 5: (Continued) A) Overview of the combined mass spectra of SUVs before and after UV irradiation in presence and absence of SUVs. The peak at a mass-to-charge ratio of m/z = 786.601 [M+H]+ corresponds to DOPC and the peak at m/z = 597.271 [M+H]+ to Ce6. B) Zoom in the peak area for DOPC (Structure I in F). Comparing the mass spectra for non-illuminated (blue) and illuminated (orange) SUVs in the absence of Ce6, one can conclude that DOPC without Ce6 is not influenced by the UV irradiation. With Ce6 after illumination, the DOPC peak is reduced. C) Zoom in the peak area for Ce6. D) Zoom in the peak area for the oxidized hydroperoxyl derivative of DOPC that emerges after the first reaction step (Structure II in F). E) Zoom in the peak area for the oxidized hydroperoxyl derivative of DOPC with both fatty acid chains oxidized that emerges after the first reaction step (Structure III in F). With adding Ce6 and irradiating the sample, both unsaturated positions in the fatty acid were transformed to hydroperoxyl groups with m/z = 850.578 [M+2O+H]+. To improve readability, the graphs were each shifted by a mass-to-charge ratio of m/z = 0.05. F) Structural formula for DOPC (Structure I), the oxidized hydroperoxyl derivative of DOPC (Structure II) and the the oxidized hydroperoxyl derivative of DOPC with both fatty acid chains oxidized (Structure III).

## 2.6 Figure S6: UV illumination dose as used for vesicle loading does not affect the functionality of bio-molecules

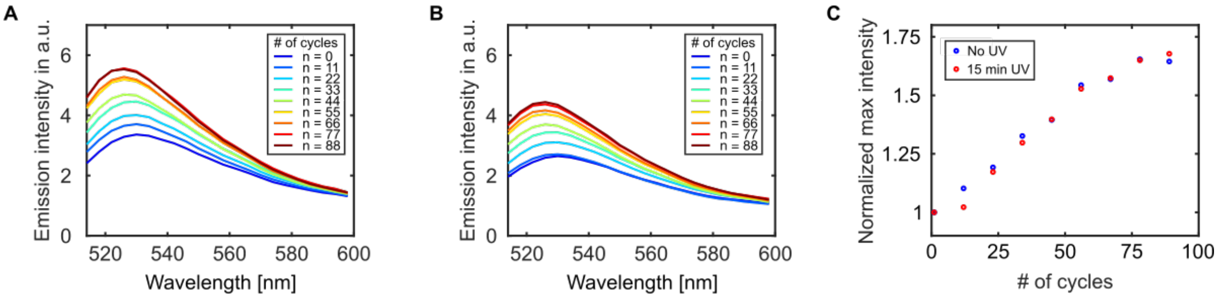


Figure 6: UV illumination dose as used for vesicle loading does not affect the functionality of bio-molecules for quantitative polymerase chain reaction (qPCR). Emission spectra (SYBR green I dye, $\lambda_{ex}$ =476 nm) of qPCR measurements without further modifications (**A**) and after 15 min of UV illumination of all components before the first PCR cycle (**B**). Note that the total intensity of the UV illuminated sample is reduced due to photobleaching of the SYBR green I dye. The control (**A**) as well as the illuminated sample (**B**) show an increase of the of the emission intensity with an increase of the number of cycles executed. In particular, the normalized maximum intensity shows the same increases at a similar rate for both conditions (**C**). This indicates that the reaction kinetics are the same for both samples. Hence, besides bleaching the UV illumination does not affect the functionality of DNA or proteins as used in qPCR.

## 2.7 Figure S7: Absorbance spectrum of Ce6

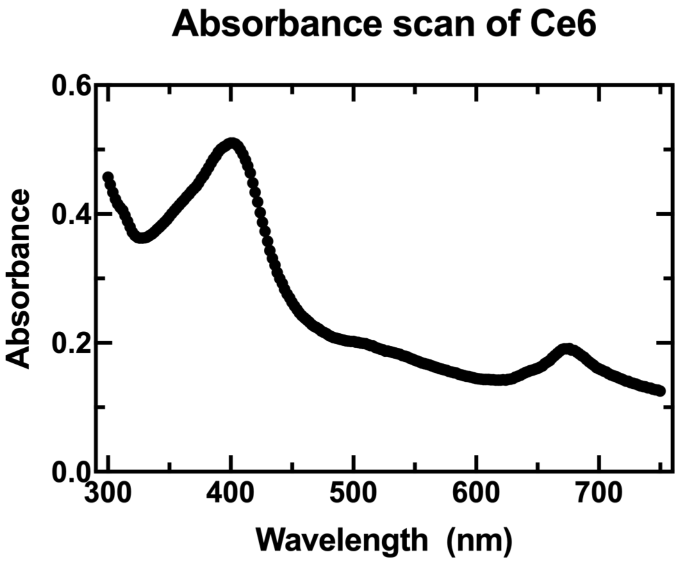


Figure 7: Absorbance spectrum of Ce6 showing absorbance across the whole range of visible light (100 µM Ce6 in 100 mM sucrose). The absorbance scan was acquired from 350-750 nm with a step size of 2 nm with a Spark plate reader from TECAN.

## 2.8 Figure S8: Autofluorescence of Ce6 prevents bleaching after division as proof for full fission

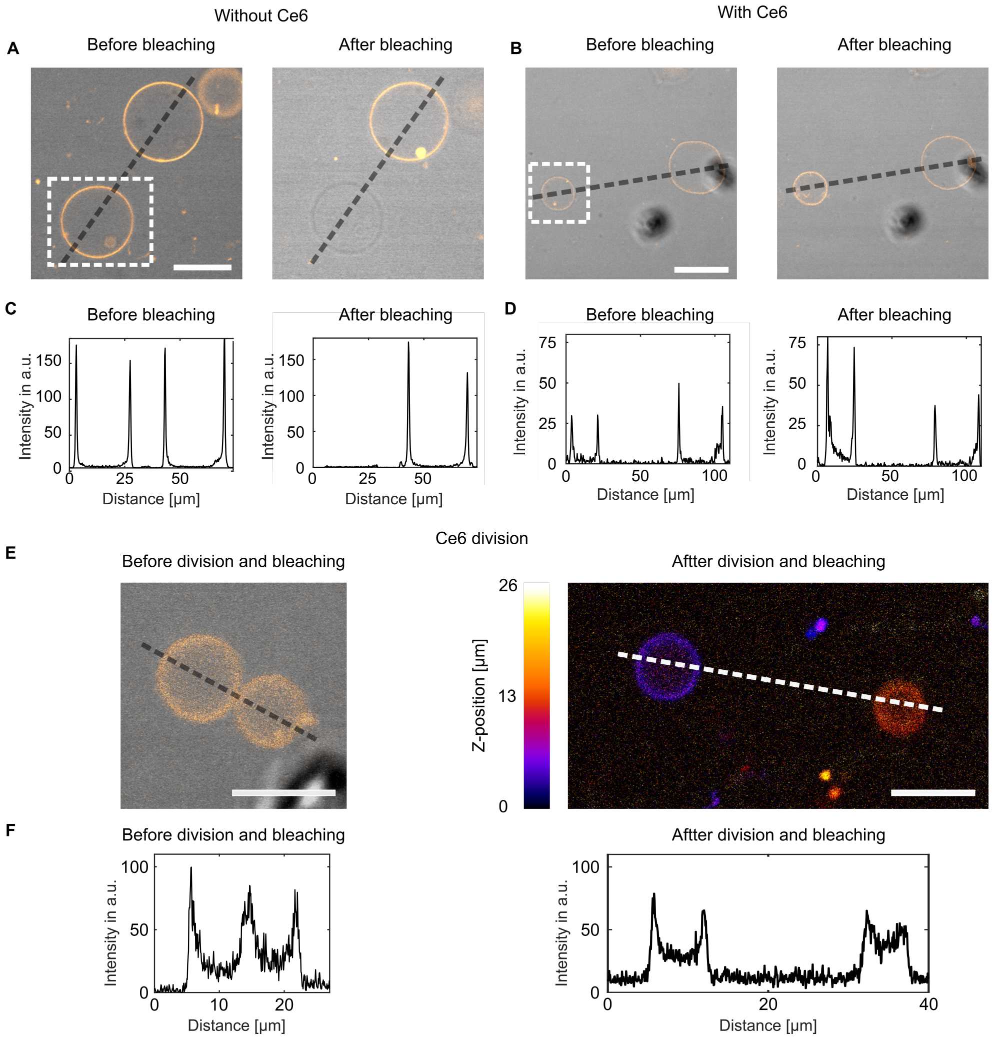


Figure 8: Autofluorescence of Ce6 prevents bleaching after division as proof for full fission. Continued on the next page.

Figure 8: (Continued) **A+B** Overlays of confocal fluorescence images ($\lambda_{ex} = 561\,nm$) and bright-field images of GUVs in the absence (**A**) and in the presence (**B**) of Ce6 (100 µM) before (left) and after (right) bleaching the area indicated by the white dotted box with 100 % laser intensity (Scale bars: 30 µm). **C+D** Intensity profiles along the gray dashed lines indicated in **A+B** in the absence (**C**) and in the presence (**D**) of Ce6 before (left) and after (right) bleaching. While the intensity of the GUV within the bleaching area is almost zero after bleaching in the absence of Ce6, it does not decrease (but even temporarily increase) when bleaching a single GUV in the presence of Ce6. We attribute this observation to the fluorescence behavior of Ce6. Full bleaching of Ce6 is most likely not possible due to constant exchange with the surrounding solution. The same behavior is observed when attempting to bleach GUVs after the Ce6 division process. **E** Overlay of a confocal fluorescence image ($\lambda_{ex} = 561\,nm$) and a bright-field image of two vesicles connected with a tight neck before division (left) and colour-coded z-projection of a confocal fluorescence z-stack of the same two vesicles after division and multiple bleaching attempts of the vesicle on the right hand side (right, scale bars: 10 µm). Note that no lipid tubes connecting the two vesicles can be detected in the entire z-stack. **F** Intensity profiles along the dashed lines as indicated in **E**.

## 2.9 Figure S9: Proof of vesicle fission

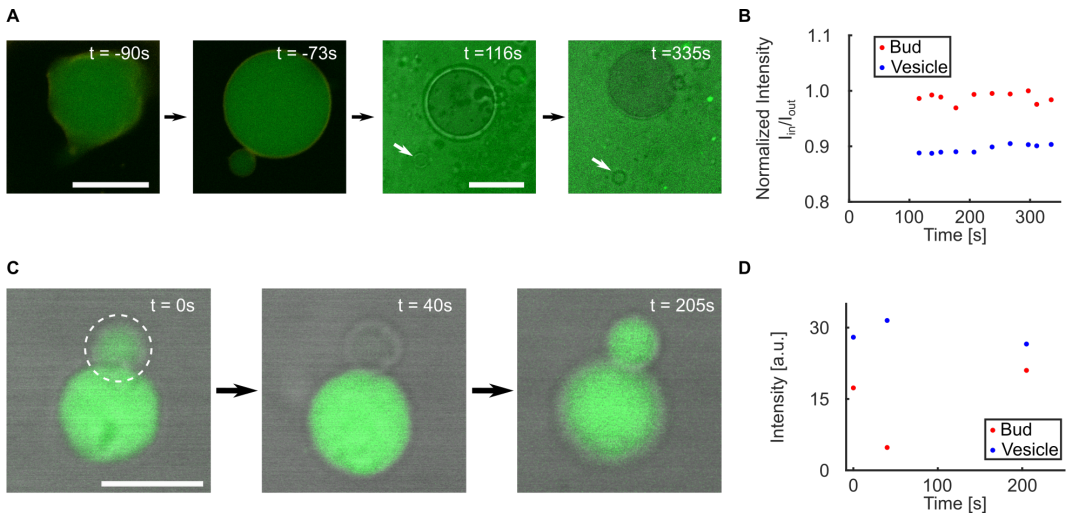


Figure 9: Proof of vesicle fission. **A** Confocal fluorescence time series of a GUV containing 10 µM Atto488-NHS ester ($\lambda_{ex}$ =488 nm) undergoing division in the presence of Ce6 and light illumination. A bud forms during 405 nm illumination of the whole vesicle and eventually detaches from the vesicle after local illumination of the neck region within 90 s. The encapsulated Atto488-NHS ester dye is bleached during the 405 nm illumination. However, at higher intensities one can observe that the vesicles drift apart (3rd and 4th image). Time points are indicated relative to the time point of fission at t = 0. Scale bars: 20 µm. **B** Normalized inner intensity of the divided vesicles shown in **A**. The fluorescence intensities of both daughter vesicles stay constant for over 200 s after division. The fluorescence intensities do not equilibrate as expected if they were still connected via a lipid nanotube. **C** Control experiment showing that transport of Atto488-NHS accross a lipid nanotube can occur and equilibration should happen within 200 s. Confocal fluorescence time series of a GUV that has a connected bud, where fluorescence recovers after photobleaching. Scale bar: 10 µm. **D** Intensities of the two vesicles in **C** before (t = 0 s), immediately after (t = 40 s) and minutes after photobleaching (t = 205 s). The fluorescence signal in the bud recovers within 200 s to its original value after photobleaching, whereas the fluorescence of the other un-bleached vesicle remains constant.

## 2.10 Figure S10: Examples of GUVs undergoing light-triggered division in the presence of Ce6

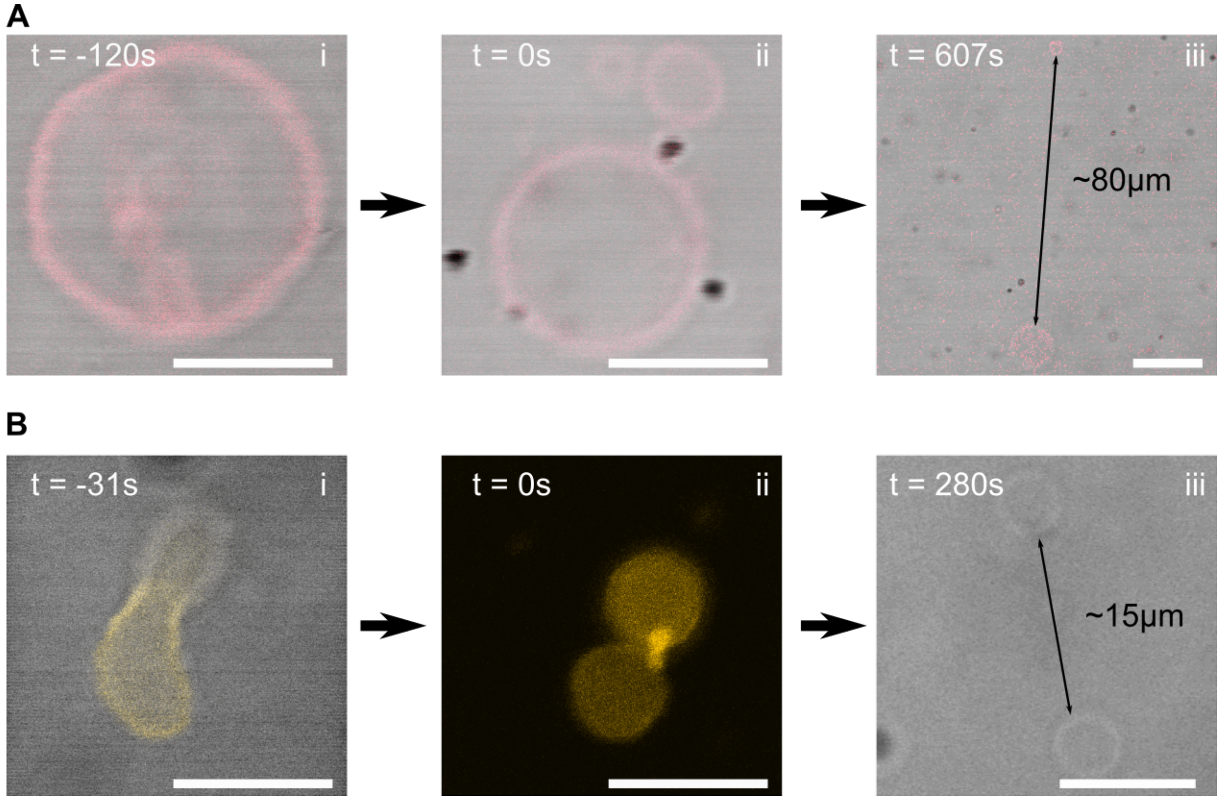


Figure 10: Examples of GUVs undergoing light-triggered division in the presence of Ce6. **A+B** Confocal fluorescence images of GUVs before (i) during (ii) and after (iii) 405 nm laser illumination. GUVs were labeled with Atto633-DOPE (**A**, $\lambda_{ex}$ =640 nm) or LissRhod PE (**B**, $\lambda_{ex}$ =561 nm). Scale bars: 10 µm (A i+ii, B i-iii) and 20 µm (A iii).

## 2.11 Figure S11: 405 nm illumination alone is not sufficient to achieve fission of deflated GUVs

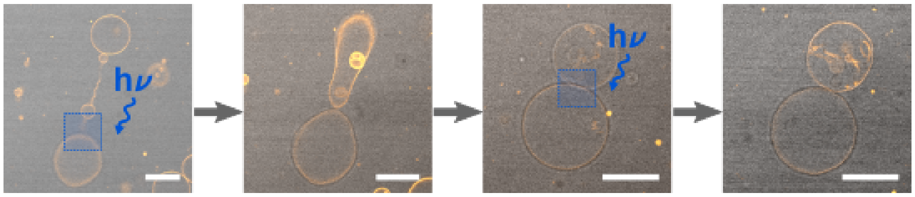


Figure 11: 405 nm illumination alone is not sufficient to achieve fission of deflated GUVs. Overlay of brightfield and confocal images of a deflated DOPC GUV (labeled with Rhodamine-PE, $\lambda_{ex} = 561$ nm) in 100 mM sucrose (reduced volume $\nu = 1.66$). Due to the excess membrane area caused by the deflation, the GUV exhibits buds which are still connected by lipid tubes. Even after repeated illumination of the interface between buds with 60 % laser intensity of a 405 nm diode laser for 3 minutes, no fission occurs without Ce6. Note that in the presence of Ce6, fission typically occurs after 10 to 50 seconds of illumination. Scale bar: 30 µm.

## 2.12 Figure S12: DNA remains intact after encapsulation into GUVs

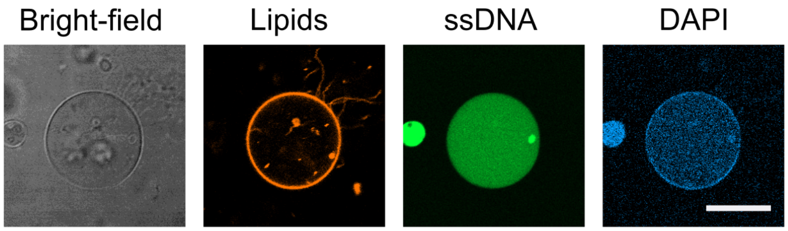


Figure 12: DNA remains intact after encapsulation into GUVs. Confocal image of a GUV (labeled with Rhodamine-PE, $\lambda_{ex} = 561\,\text{nm}$) containing fluorescently labeled single-stranded DNA (ssDNA, labeled with Atto488, $\lambda_{ex} = 488\,\text{nm}$) after addition of membrane-permeable DAPI DNA stain ($\lambda_{ex} = 405\,\text{nm}$). The successful staining of the DNA with DAPI indicates that the DNA remains intact inside GUVs. Scale bar: 20 µm.

## 2.13 Figure S13: DNA remains encapsulated in the daughter vesicles after Ce6-mediated division.

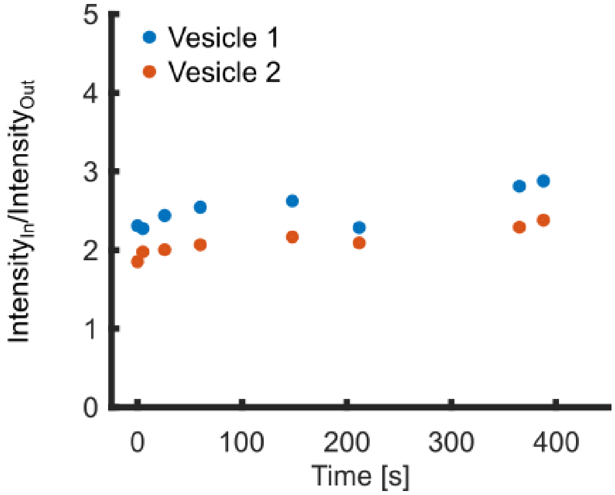


Figure 13: Quantification of the fluorescence intensity of the DNA in the daughter compartments confirms that DNA stays encapsulated after division. Normalized intensity inside both daughter vesicles over time after fission occurred. Note that before initiation of the division process the ratio of the intensity inside over the intensity outside the vesicles was around $I_{\mathrm{in}}/I_{\mathrm{out}} \approx 25$. The significant drop of the inner intensity is due to bleaching by the 405 nm illumination necessary for division. After division the inner intensity stays constant in both daughter vesicles demonstrating that the DNA content stays encapsulated after division.

# 3 Supporting Notes

## 3.1 Supporting Note 1: Ce6-mediated volume shrinkage

As we observe a drastic shrinkage (75% volume loss) of the GUVs when they are exposed to UV light or illumination with a 405 nm laser over longer time scales (tens of minutes), we hypothesize that at least one of the following four mechanisms occurs:

1) Membrane area reduction via formation of buds or lipid tubes.

2) Peroxidation of both lipid tails makes the DOPC lipids hydrophilic enough to be excluded from the membrane into the aqueous phase.

3) Peroxidation ultimately leads to cleavage of lipid tails with subsequent exclusion of cleavage products from the membrane.

4) The increase of spontaneous curvature via lipid peroxidation leads to the formation and fission of small vesicles ($\leq 100$ nm).

Small buds and lipid tubes can be hard to detect with confocal fluorescence microscopy, however, an amount of lipid tubes that leads to such a drastic area and volume reduction should, in principle, be visible in confocal images. Thus, Hypothesis 1 seems unlikely. We tried to test Hypothesis 2 via mass spectrometry of the aqueous phase (without lipid vesicles) after vesicles were subjected to UV light in the presence of Ce6. However, no significant signal of the peroxidized lipids in the supernatant could be detected. Furthermore, we carried out additional mass spectrometry measurements of the vesicle-containing solution to verify lipid peroxidation and to test for the presence of cleavage products. While Hypothesis 3 has been proposed in previous publications[3,4], we were not able to detect the proposed cleavage products in mass spectrometry experiments. Due to the large volume decrease, we do not expect that the fraction of the cleavage products falls below the detection limit. Hypothesis 4 remains viable. Small vesicles would fall below the resolution limit of the confocal microscope and since we observe vesicle division after deflation, it is conceivable that fission of smaller buds occurs under the conditions used for pore formation. Nevertheless, it is possible that a

combination of multiple of the four hypothesis contribute to variable extends to the observed volume reduction.

## 3.2 Supporting Note 2: Statistics and reproducibility of the division mechanism

Table 1 below is summarizing the number of repeats of the experiment and the controls.

Table 1: Summary of the total number of repeats for the experiment and controls ($\mathrm{N_{tot}}$) and the number of respective fission events ($\mathrm{N_F}$). Note that we only counted fission events where we observed the entire division process.

| Name | Condition | Total number of repeats | Number of complete fission events |
|---|---|---|---|
| Experiment (E) | +Ce6 / +Deflation | $\mathrm{N_{tot,E}} = 200$ | $\mathrm{N_{F,E}} = 6$ |
| Control 1 (C1) | -Ce6 / +Deflation | $\mathrm{N_{tot,C1}} = 150$ | $\mathrm{N_{F,C1}} = 0$ |
| Control 2 (C2) | +Ce6 / -Deflation | $\mathrm{N_{tot,C2}} = 150$ | $\mathrm{N_{F,C2}} = 0$ |

From these results, we obtain a probability for the completely observed fission processes of $P_{\mathrm{F,E}} = \frac{N_{\mathrm{F,E}}}{N_{\mathrm{tot,E}}} = 6/200 = 0.03$. Note that this is a conservative estimate as we only included divisions that we observed from the start to the end. It does not including those vesicles that were already in a dumbbell shape – if we did include them the success rate would raise to 10.5 %. Note that such events were not observed in the controls. From this, we can calculate the probability $P$ for not observing division in the control experiments (assuming a 3 % probability for fission events, $\mathrm{P_{F,E}} = 0.03$) as follows:

$$P(N_{\mathrm{F,C1}} = 0 | P_{\mathrm{F,C1}} = P_{\mathrm{F,E}}) = (1 - 0.03)^{150} = 0.010 \equiv 1.0\,\% \quad (1)$$

whereby $\mathrm{N}_{F,C1}$ is the number of fission events observed in Control 1, which was repeated 150 times. The same result holds true for Control 2. When taking both controls together, with $\mathrm{N_{tot,C1}} + \mathrm{N_{tot,C2}} = 300$, the combined probability of seeing fission in none of them is

$$P(N_{\mathrm{F,C}} = 0 | P_{\mathrm{F,C}} = P_{\mathrm{F,E}}) = (1 - 0.03)^{300} = 0.01\,\% \tag{2}$$

If we include the cases where the vesicles were already in a dumbbell shape before illumination and calculate with an increased probability of 10.5 % for a fission event ($\mathrm{P}_{\mathrm{F,E}} = 0.105$), we find that the probability for observing no fission in 300 controls becomes extremely low:

$$P(N_{\mathrm{F,C}} = 0 | P_{\mathrm{F,C}} = P_{\mathrm{F,E}}) = (1 - 0.105)^{300} = 3.5 \cdot 10^{-15} \tag{3}$$

Taken together, we can thus be very confident that division is indeed caused by illumination in the presence of Ce6.

# 4 Supporting Videos

## 4.1 Supporting Video 1: Ce6-mediated division of GUVs

Ce6-mediated division of GUVs and respective controls. Confocal time series of a GUV (orange, DOPC lipids, labeled with 1 % Rhodamine-PE, $\lambda_{ex}$ =561 nm) undergoing the Ce6-mediated division process during 405 nm illumination and control experiments in presence or absence of Ce6. Only for the dividing vesicle in presence of Ce6 and deflation, the GUV morphology changes from a prolate to a dumbbell shape and subsequent illumination of the neck region leads to full division of the two compartments (see Figure 3, main text).

## 4.2 Supporting Video 2: Ce6-mediated division of DNA-containing GUVs

Ce6-mediated division of DNA-containing GUVs. Confocal time series of a GUV (orange, DOPC lipids, labeled with Rhodamine-PE, $\lambda_{ex}$ =561 nm) containing 5 µM single-stranded

DNA (blue, labeled with Atto488, $\lambda_{ex}$ =488 nm) undergoing the Ce6-mediated division process during 405 nm illumination. The GUV morphology changes from a prolate to a dumbbell shape until complete neck fission occurs after 52 s of illumination.

# References


(1) Angelova, M. I.; Dimitrov, D. S. Liposome electroformation. *Faraday Discussions of the Chemical Society* **1986**, *81*, 303.

(2) Dreher, Y.; Jahnke, K.; Bobkova, E.; Spatz, J. P.; Göpfrich, K. Division and Regrowth of Phase-Separated Giant Unilamellar Vesicles**. *Angewandte Chemie International Edition* **2021**, *60*, 10661–10669.

(3) Cwiklik, L.; Jungwirth, P. Massive oxidation of phospholipid membranes leads to pore creation and bilayer disintegration. *Chemical Physics Letters* **2010**, *486*, 99–103.

(4) Bour, A.; Kruglik, S. G.; Chabanon, M.; Rangamani, P.; Puff, N.; Bonneau, S. Lipid Unsaturation Properties Govern the Sensitivity of Membranes to Photoinduced Oxidative Stress. *Biophysical Journal* **2019**, *116*, 910–920.
